# 3D MR elastography at 0.55 T: Concomitant field effects and feasibility

**DOI:** 10.1002/mrm.30377

**Published:** 2024-11-25

**Authors:** Omar Isam Darwish, Pierluigi Di Cio, Ralph Sinkus, Radhouene Neji

**Affiliations:** ^1^ Research Department of Imaging Physics and Engineering, School of Biomedical Engineering and Imaging Sciences King's College London London UK; ^2^ MR Predevelopment Siemens Healthineers AG Erlangen Germany; ^3^ INSERM U1148, LVTS University Paris Diderot Paris France

**Keywords:** concomitant fields, Hadamard encoding, liver, low field MR, MR elastography

## Abstract

**Purpose:**

To demonstrate the feasibility of hepatic 3D MR elastography (MRE) at 0.55 T in healthy volunteers using Hadamard encoding and to study the effects of concomitant fields in the domain of MRE in general.

**Methods:**

Concomitant field effects in MRE are assessed using a Taylor series expansion and an encoding scheme is proposed to study the corresponding effects on 3D MRE at 0.55 T in numerical simulations and in phantom experiments. In addition, five healthy volunteers were enrolled and scanned at 60 Hz mechanical excitation with a Hadamard‐encoded 3D MRE sequence at 0.55 T and were also scanned with a reference 3D MRE sequence at 3 T for comparison. The retrieved biomechanical parameters were the magnitude of the complex shear modulus (|G*|), the shear wave speed (Cs), and the loss modulus (*G*″). Comparison of apparent SNR between 3 T and 0.55 T was performed.

**Results:**

Theoretical analysis, numerical simulations and phantom experiments demonstrated that the effects of concomitant fields in 3D MRE at 0.55 T are negligible. In the healthy volunteer experiments, the mean values of |G*|, Cs, and G″ in the liver were 2.1 ± 0.3 kPa, 1.5 ± 0.1 m/s, and 0.8 ± 0.1 kPa at 0.55 T, respectively, and 2.0 ± 0.2 kPa, 1.5 ± 0.1 m/s, and 0.9 ± 0.1 kPa at 3 T, respectively. Bland–Altman analysis demonstrated good agreement between the biomechanical parameters retrieved at 0.55 T and 3 T. A 2.1‐fold relative apparent SNR decrease was observed in 3D MRE at 0.55 T in comparison with 3 T.

**Conclusion:**

Hepatic 3D MRE is feasible at 0.55 T, showing promising initial results in healthy volunteers.

## 1 INTRODUCTION

Hepatic 3D MR elastography (3D MRE, where “3D” refers to three‐dimensional motion encoding) has shown promises for measuring liver fibrosis and grading liver inflammation using viscoelastic parameters derived from the 3D displacement field.^1^, ^2^ Fibrosis is staged using the shear wave speed (Cs [m/s]) and inflammation is graded with the loss modulus (*G*″ [kPa]).^3^

To our knowledge, hepatic 3D MRE has been limited to high field MR systems (B_0_ ≥1.5 T). Nonetheless, expanding hepatic 3D MRE to wide‐bore low field MR systems (B_0_ ≤1.0 T) can serve as a means of accommodating liver patients with high body mass index (BMI) and/or claustrophobia such as non‐alcoholic fatty liver disease (NAFLD) patients. NAFLD is a growing global healthy crisis and enabling access to 3D MRE for NAFLD patients is becoming of importance in the clinic.^4^ Furthermore, the longer T_2_* relaxation times at low field may be beneficial to mitigate iron overload in NAFLD patients.^5^, ^6^ Another aspect to consider is that low field MR systems bring down the financial entry point of MR, which might allow a wider spread of hepatic 3D MRE in middle‐income countries.^7^

However, low field MR systems come with a penalty in SNR, which is directly proportional to a penalty in phase‐to‐noise ratio (PNR) in 3D MRE.^8^ The SNR and PNR penalty associated with low field MR has led to very limited use of MRE at field strengths below 1.0 T, with very few studies focusing on phantoms, and ex vivo tissue,^9^, ^10^ and one in vivo study proposing a fast MRE acquisition technique at 0.1 T for assessment of shear stiffness in the human skeletal muscle.^11^

A Hadamard motion encoding scheme may be used to inherently increase the number of averages,^12^ thereby mitigating the decrease in PNR. Hadamard encoding has been used for obtaining FIDs from different regions in spectroscopic imaging,^13^ as well as for chemical shift imaging of thin slabs using cascaded slice excitation with good SNR and slice profile and without point spread function degradation.^14^, ^15^, ^16^ Recent work has introduced Hadamard motion encoding in MRE and shown improvement in displacement‐to‐noise ratio compared to conventional motion encoding schemes and shown applications in hepatic MRE.^2^, ^8^, ^12^, ^17^

Hadamard motion encoding applies unique combinations of motion encoding gradients on all the gradient axes simultaneously; simultaneous longitudinal and transverse gradients lead, however, to additional concomitant field terms and these fields are inversely proportional to the static magnetic field B_0_,^18^ thereby becoming more pronounced at low field MR systems (B_0_ ≤1.0 T).

This study is organized as follows: we first consider a general MRE experiment and study theoretically the effects of concomitant fields because of motion encoding gradients in MRE. We then evaluate these effects in numerical simulations and in phantom experiments in the special case of Hadamard‐encoded MRE and demonstrate feasibility of hepatic 3D MRE at 0.55 T in healthy volunteer experiments.

### 1.1 Theory

A magnetic gradient field realizes a spatially linear variation of the magnetic field. In free space, the magnetic field resulting from applying a linear magnetic gradient has to obey the curl‐free and divergence‐free conditions of the Maxwell equations. It has been shown that this leads to concomitant gradient fields (also known as Maxwell terms) coexisting with the applied linear gradient field.^18^ These concomitant fields lead to additional phase accumulation errors in phase‐contrast MRI experiments.^18^

When applying a magnetic gradient field, there is a concomitant magnetic field $B_{c}$ resulting from Maxwell's equations and given to the lowest order by the following equation^18^:

(1) $$B_{c}\left(x,y,z,t\right)=\frac{1}{2B_{0}}\left(G_{x}^{2}z^{2}+G_{y}^{2}z^{2}+G_{z}^{2}\frac{x^{2}+y^{2}}{4}-G_{x}\;G_{z}\mathrm{xz}-G_{y}\;G_{z}\mathrm{yz}\right),$$

where $B_{0}$ is the static field, $G_{x},G_{y},$ and $G_{z}$ are the applied magnetic gradients at time point t, and $x_{,\;}y,$ and z are the spatial coordinates given in the physical coordinate system of the scanner. Please note that $B_{c}$ is composed of self‐squared terms (such as the term in $G_{x}^{2}$) and cross‐terms (such as the term in $G_{x}\;G_{z}$)^18^ the latter appear only if gradients are applied simultaneously in z and another perpendicular direction (x or y), which is the case for Hadamard encoding.

Let us consider a bipolar motion encoding gradient G with lobes 1 and 2 $(G_{2}=-G_{1}$), with a total duration *T*, applied on $x_{,\;}\;y,$ and *z* without overlap with any imaging gradient in the MRE sequence.

The phase accrual $\varphi_{c}$ because of $B_{c}$ by a spin isochromat moving from position $\left(x_{1},y_{1},z_{1}\right)$ during lobe 1 to position $\left(x_{2},y_{2},z_{2}\right)$ during lobe 2 is given by:

(2) $$\varphi_{c}=\varphi_{1}+\varphi_{2},$$

(3) $$\varphi_{1}=\frac{\gamma T}{4B_{0}}\left(G_{x}^{2}z_{1}^{2}+G_{y}^{2}z_{1}^{2}+G_{z}^{2}\frac{x_{1}^{2}+y_{1}^{2}}{4}-G_{x}G_{z}x_{1}z_{1}-G_{y}G_{z}y_{1}z_{1}\right),$$

(4) $$\varphi_{2}=\frac{\gamma T}{4B_{0}}\left(G_{x}^{2}z_{2}^{2}+G_{y}^{2}z_{2}^{2}+G_{z}^{2}\frac{x_{2}^{2}+y_{2}^{2}}{4}-G_{x}G_{z}x_{2}z_{2}-G_{y}G_{z}y_{2}z_{2}\right),$$

where $\varphi_{1}$ is the phase accrual during lobe 1 and $\varphi_{2}$ is the phase accrual during lobe 2 because of $B_{c}$. Please note that the above formulation assumes that the position of the spin isochromat is constant per motion encoding gradient lobe, which is a reasonable assumption if the motion encoding gradient duration (*T*) is very short.

Defining the displacement $\left(\mu_{x},\mu_{y},\mu_{z}\right)$ as $\left(x_{2}-x_{1},y_{2}-y_{1},z_{2}-z_{1}\right)$, which in hepatic MRE applications is very small (˜200μm),^19^ allows us to rewrite $\varphi_{2}$ using Taylor's expansion:

(5) $$\begin{array}{cc} \varphi_{2}\approx \varphi_{1} & +\frac{\gamma T}{2}\;\left(\mu_{x}\frac{\partial B_{c}\left(x_{1},y_{1},z_{1}\right)}{\partial x}+\mu_{y}\frac{\partial B_{c}\left(x_{1},y_{1},z_{1}\right)}{\partial y}\right.\\ & \;\left.+\;\mu_{z}\frac{\partial B_{c}\left(x_{1},y_{1},z_{1}\right)}{\partial z}\right). \end{array}$$

This leads to the following equations:

(6) $$\begin{array}{cc} \varphi_{2} & \approx \varphi_{1}+\mu_{x}\frac{\gamma T}{4B_{0}}\left({2G}_{z}^{2}\frac{x_{1}}{4}-G_{x}\;G_{z}z_{1}\right)\\ & \;+\mu_{y}\frac{\gamma T}{4B_{0}}\left({2G}_{z}^{2}\frac{y_{1}}{4}-G_{y}\;G_{z}z_{1}\right)\\ & \;+\mu_{z}\frac{\gamma T}{4B_{0}}\left({2G}_{x}^{2}z_{1}-{2G}_{y}^{2}z_{1}-G_{x}\;G_{z}x_{1}-G_{y}\;G_{z}y_{1}\right), \end{array}$$

(7) $$\varphi_{2}\approx \varphi_{1}+\varphi_{c,\text{dynamic}},$$

where $\varphi_{c,\text{dynamic}}$ is the phase accrual because of $B_{c}$ that is related to the displacement $\left(\mu_{x},\mu_{y},\mu_{z}\right).$

Recall that $\varphi_{c}=\varphi_{1}+\varphi_{2}$, which can then be written as:

(8) $$\varphi_{c}=2\;\varphi_{1}+\varphi_{c,\text{dynamic}}=\varphi_{c,\text{static}}+\varphi_{c,\text{dynamic}},$$

where $\varphi_{c,\text{static}}$ is the phase accrual $\left(2\;\varphi_{1}\right)$ because of $B_{c}$, which is dependent on the position $\left(x_{1},y_{1},z_{1}\right)$. We also provide the full analytical solution of $\varphi_{c}$ for a spin isochromat undergoing an ideal periodic sinusoidal motion (shown in the Material S1).

Moreover, the classical phase accrual $\varphi_{\mathrm{enc}}$ because of the motion encoding gradients by the spin isochromat moving from position $\left(x_{1},y_{1},z_{1}\right)$ during lobe 1 to position $\left(x_{2},y_{2},z_{2}\right)$ during lobe 2 related to the displacement $\left(\mu_{x},\mu_{y},\mu_{z}\right)$ is given by:

(9) $$\varphi_{\mathrm{enc}}=\frac{\gamma T}{2}\left(G_{x}\mu_{x}+G_{y}\mu_{y}+G_{z}\mu_{z}\right).$$

Comparing the terms of $\varphi_{\mathrm{enc}}$ with the terms of $\varphi_{c,\text{dynamic}}$ demonstrates that the additional terms because of $B_{c}$ that are related to the displacement $\left(\mu_{x},\mu_{y},\mu_{z}\right)$ are negligible. For example, when comparing the terms in $\mu_{x}$, because $G_{x}$, and $G_{z}$ are of the order of 10 to 20 mT/m, $B_{0}$ is 0.55 T, $x_{1}$ is of the order of 100 to 200 mm, and $z_{1}$ is of the order of 10 to 20 mm we obtain the following result:

(10) $$\begin{array}{cc} & \frac{\frac{\gamma T}{2}G_{x}\mu_{x}}{\frac{\gamma T}{4B_{0}}\left({2G}_{z}^{2}\frac{x_{1}}{4}-G_{x}\;G_{z}z_{1}\right)\mu_{x}}˜10^{2}\Rightarrow \frac{\gamma T}{2}G_{x}\mu_{x}\\ & \;\gg \frac{\gamma T}{4B_{0}}\left({2G}_{z}^{2}\frac{x_{1}}{4}-G_{x}\;G_{z}z_{1}\right)\mu_{x}, \end{array}$$

where ˜ indicates an order of magnitude. Consequently, neglecting $\varphi_{c,\text{dynamic}}$ leaves us with $\varphi_{c,\text{static}}$ only:

(11) $$\varphi_{c,\text{static}}=\frac{\gamma T}{2B_{0}}\left(G_{x}^{2}z_{1}^{2}+G_{y}^{2}z_{1}^{2}+G_{z}^{2}\frac{x_{1}^{2}+y_{1}^{2}}{4}-G_{x}G_{z}x_{1}z_{1}-G_{y}G_{z}y_{1}z_{1}\right),$$

3D MRE acquisitions include several mechanical wave phase offsets, usually four mechanical wave phase offsets, to sample the mechanical wave at different time points. We now analyze the changes of $\varphi_{c,\text{static}}$ between two wave phase offsets wp1, and wp2, to investigate whether $\varphi_{c,\text{static}}$ depends on the wave phase offset.

Let us consider a spin isochromat that moved from position $\left(x_{\mathrm{wp}1},y_{\mathrm{wp}1},z_{\mathrm{wp}1}\right)$ at the first wave phase offset wp1 to $\left(x_{\mathrm{wp}2},y_{\mathrm{wp}2},z_{\mathrm{wp}2}\right)$ at the second wave phase offset wp2. Let $\varphi_{c,\text{static},\mathrm{wp}1}$ and $\varphi_{c,\text{static},\mathrm{wp}2}$ be the $\varphi_{c,\text{static}}$ during wave phase offsets wp1 and wp2, respectively. Similarly to Eqs. (3) and (4), $\varphi_{c,\text{static},\mathrm{wp}1}$ and $\varphi_{c,\text{static},\mathrm{wp}2}$ are then given by the following equations:

(12) $$\begin{array}{cc} & \varphi_{c,\text{static},\mathrm{wp}1}=\frac{\gamma T}{2B_{0}}\left(G_{x}^{2}z_{\mathrm{wp}1}^{2}+G_{y}^{2}z_{\mathrm{wp}1}^{2}+G_{z}^{2}\frac{x_{\mathrm{wp}1}^{2}+y_{\mathrm{wp}1}^{2}}{4}\right.\\ & \;\left.-G_{x}G_{z}x_{\mathrm{wp}1}z_{\mathrm{wp}1}-G_{y}G_{z}y_{\mathrm{wp}1}z_{\mathrm{wp}1}\right), \end{array}$$

(13) $$\begin{array}{cc} & \varphi_{c,\text{static},\mathrm{wp}2}=\frac{\gamma T}{2B_{0}}\left(G_{x}^{2}z_{\mathrm{wp}2}^{2}+G_{y}^{2}z_{\mathrm{wp}2}^{2}+G_{z}^{2}\frac{x_{\mathrm{wp}2}^{2}+y_{\mathrm{wp}2}^{2}}{4}\right.\\ & \;\left.-G_{x}G_{z}x_{\mathrm{wp}2}z_{\mathrm{wp}2}-G_{y}G_{z}y_{\mathrm{wp}2}z_{\mathrm{wp}2}\right). \end{array}$$

We note that the relative displacement between the wave phase offsets $\left(x_{\mathrm{wp}2}-x_{\mathrm{wp}1},y_{\mathrm{wp}2}-y_{\mathrm{wp}1},z_{\mathrm{wp}2}-z_{\mathrm{wp}1}\right)$ is again very small in hepatic 3D MRE applications (˜200μm) allowing us to rewrite $\varphi_{c,\text{static},\mathrm{wp}2}$ using Taylor's expansion similar to the previous expansion in Eq. (5):

(14) $$\begin{array}{cc} & \varphi_{c,\text{static},\mathrm{wp}2}\approx \varphi_{c,\text{static},\mathrm{wp}1}\\ & +\left\{\left(x_{\mathrm{wp}2}-x_{\mathrm{wp}1}\right)\frac{\partial \varphi_{c,\text{static}}\left(x_{\mathrm{wp}1},y_{\mathrm{wp}1},z_{\mathrm{wp}1}\right)}{\partial x}\right.\\ & +\left(y_{\mathrm{wp}2}-y_{\mathrm{wp}1}\right)\frac{\partial \varphi_{c,\text{static}}\left(x_{\mathrm{wp}1},y_{\mathrm{wp}1},z_{\mathrm{wp}1}\right)}{\partial y}\\ & \left.+\left(z_{\mathrm{wp}2}-z_{\mathrm{wp}1}\right)\frac{\partial \varphi_{c,\text{static}}\left(x_{\mathrm{wp}1},y_{\mathrm{wp}1},z_{\mathrm{wp}1}\right)}{\partial z}\right\}, \end{array}$$

(15) $$\begin{array}{cc} & \varphi_{c,\text{static},\mathrm{wp}2}\approx \varphi_{c,\text{static},\mathrm{wp}1}\\ & +\frac{\gamma T}{2B_{0}}\;\left\{\;\left(x_{\mathrm{wp}2}-x_{\mathrm{wp}1}\right)\left({2G}_{z}^{2}\frac{x_{\mathrm{wp}1}}{4}-G_{x}\;G_{z}z_{\mathrm{wp}1}\right)\right.\\ & +\left(y_{\mathrm{wp}2}-y_{\mathrm{wp}1}\right)\left({2G}_{z}^{2}\frac{y_{\mathrm{wp}1}}{4}-G_{y}\;G_{z}z_{\mathrm{wp}1}\right)\\ & \left.+\left(z_{\mathrm{wp}2}-z_{\mathrm{wp}1}\right)\left({2G}_{x}^{2}z_{\mathrm{wp}1}-{2G}_{y}^{2}z_{\mathrm{wp}1}-G_{x}\;G_{z}x_{\mathrm{wp}1}-G_{y}\;G_{z}y_{\mathrm{wp}1}\right)\right\}. \end{array}$$

Furthermore, the variation of $\varphi_{\mathrm{enc}}$ because of the motion encoding gradients between the two wave phase offsets wp1 and wp2 is given by:

(16) $$\;\frac{\gamma T}{2}\left(\left(\mu_{x,\mathrm{wp}2}-\mu_{x,\mathrm{wp}1}\right)G_{x}+\left(\mu_{y,\mathrm{wp}2}-\mu_{y,\mathrm{wp}1}\right)G_{y}+\left(\mu_{z,\mathrm{wp}2}-\mu_{z,\mathrm{wp}1}\right)G_{z}\right).$$

Where $\mu_{x,\mathrm{wp}1}$, $\mu_{y,\mathrm{wp}1}$, $\mu_{z,\mathrm{wp}1}$ (respectively, $\mu_{x,\mathrm{wp}2}$, $\mu_{y,\mathrm{wp}2}$, $\mu_{z,\mathrm{wp}2}$) represent the displacements at wave phase offset wp1 (respectively, wp2).

We note that the variation of $\varphi_{c,\text{static}}$ between the wave phase offsets is small compared to the variation of $\varphi_{\mathrm{enc}}$. For example, because $G_{x}$, and $G_{z}$ are of the order of 10 to 20 mT/m, $B_{0}$ is 0.55 T, $x_{\mathrm{wp}1}$ is of the order of 100 to 200 mm, and $z_{\mathrm{wp}1}$ is of the order of 10 to 20 mm, and considering that $\left(x_{\mathrm{wp}2}-x_{\mathrm{wp}1}\right)$ and $\left(\mu_{x,\mathrm{wp}2}-\mu_{x,\mathrm{wp}1}\right)$ have the same order of magnitude, which is a reasonable assumption for a motion encoding gradient frequency close to the mechanical vibration frequency (details are provided in Material S2), we obtain the following:

(17) $$\begin{array}{cc} & \frac{\;\frac{\gamma T}{2}\left(\mu_{x,\mathrm{wp}2}-\mu_{x,\mathrm{wp}1}\right)G_{x}}{\frac{\gamma T}{2B_{0}}\left(x_{\mathrm{wp}2}-x_{\mathrm{wp}1}\right)\left({2G}_{z}^{2}\frac{x_{\mathrm{wp}1}}{4}-G_{x}\;G_{z}z_{\mathrm{wp}1}\right)}˜10^{2}\\ & \;\Rightarrow \frac{\gamma T}{2}\left(\mu_{x,\mathrm{wp}2}-\mu_{x,\mathrm{wp}1}\right)G_{x}\gg \frac{\gamma T}{2B_{0}}\left(x_{\mathrm{wp}2}-x_{\mathrm{wp}1}\right)\\ & \;\left({2G}_{z}^{2}\frac{x_{\mathrm{wp}1}}{4}-G_{x}\;G_{z}z_{\mathrm{wp}1}\right). \end{array}$$

Therefore, $\varphi_{c,\text{static}}$ can be considered to be constant through the different wave phase offsets.

We can conclude from the derivations above that there are no additional corrections needed to account for the effects of concomitant fields in hepatic 3D MRE as the phase accrual contributions that are related to displacement $\left(\varphi_{c,\text{dynamic}}\right)$ are negligible and the remaining phase accrual $\left(\varphi_{c,\text{static}}\right)$ is filtered out by the temporal Fourier transform applied in MRE reconstruction because it contributes only to the DC component of the Fourier transform. We assumed that the motion encoding lobes were of very short duration, however, by dividing each lobe in several, short time intervals, the assumption is valid in each of these intervals and the results obtained above still hold via a summation of the phase accruals across these time intervals. For example, having shown that $\varphi_{\mathrm{enc}}\gg$ $\varphi_{c,\text{dynamic}}$ leads to $\sum \varphi_{\mathrm{enc}}$ ≫ $\sum \varphi_{c,\text{dynamic}}$ where ∑ represents the summation over the short time intervals.

## 2 METHODS

### 2.1 The effect of concomitant fields on Hadamard‐encoded 3D MRE

To design an experiment that measures the effect of concomitant fields on Hadamard‐encoded 3D MRE, we first rewrite Eq. (11) following the terminology in^18^ as follows:

(18) $$\varphi_{c,\text{static}}=\frac{\gamma T}{2B_{0}}\left(\overset{\text{self}-\text{squared terms}}{\overbrace{G_{x}^{2}z^{2}+G_{y}^{2}z^{2}+G_{z}^{2}\frac{x^{2}+y^{2}}{4}}}\underset{\text{cross terms}}{\underbrace{-G_{x}\;G_{z}\mathrm{xz}-G_{y}\;G_{z}\mathrm{yz}}}\right).$$

An Hadamard‐encoded MRE measurement consists of unique combinations of motion encoding gradients applied on all gradient axes simultaneously with equal amplitudes.^12^ The following 4 × 4 Hadamard matrix (H) is typically used in MRE measurement:

(19) $$H=\left[\begin{array}{llll} -\;1 & +1 & -1 & +1\\ +1 & -1 & -1 & +1\\ -\;1 & -1 & +1 & +1\\ +1 & +1 & +1 & +1 \end{array}\right].$$

The first three terms in Eq. (18), the self‐squared terms, are constant throughout the different measurements of Hadamard encoding and are, therefore, encoded in the same term that encodes constant phase errors such as magnetic field inhomogeneity and motion encoding because of the imaging gradients. However, the last two terms, the cross terms, change signs between the different measurements of Hadamard encoding depending on the polarity of the applied motion encoding gradients. As a result, the cross terms cannot be resolved using a conventional 4 × 4 Hadamard encoding matrix. To resolve the cross terms we extend Hadamard encoding to a 6 × 6 encoding matrix M that accounts for the different possible sign combinations of the cross terms:

(20) $$M=\left[\begin{array}{llllll} -\;1 & +1 & -1 & +1 & -1 & +1\\ +1 & -1 & -1 & +1 & -1 & -1\\ -\;1 & -1 & +1 & +1 & +1 & -1\\ +1 & +1 & +1 & +1 & +1 & +1\\ +1 & -1 & +1 & +1 & -1 & +1\\ -\;1 & +1 & +1 & +1 & -1 & -1 \end{array}\right],$$

(21) $$\left[\begin{array}{l} m_{1}\\ m_{2}\\ m_{3}\\ m_{4}\\ m_{5}\\ m_{6} \end{array}\right]=M\left[\begin{array}{l} \varphi_{U_{z}}\\ \varphi_{U_{x}}\\ \varphi_{U_{y}}\\ \varphi_{\mathrm{err}}\\ \varphi_{\mathrm{xz}}\\ \;\varphi_{\mathrm{yz}} \end{array}\right],$$

where $m_{k}$ (*k* = 1, 2, …, 6) is the k‐th MRE phase measurement, $\varphi_{U_{x}},\varphi_{U_{y}},$ and $\varphi_{U_{z}}$ are the phase accruals because of the 3D displacement field in the scanner physical coordinates x,y, and z, respectively. $\varphi_{\mathrm{err}}$ is the phase accrual because of constant phase errors such as magnetic field inhomogeneity, motion encoding because of the imaging gradients and self‐squared terms. $\varphi_{\mathrm{xz}}$ and $\varphi_{\mathrm{yz}}$ are the phase accrual because of the first and second cross terms, respectively.

### 2.2 Numerical simulations

A numerical simulation was performed to investigate the effects of concomitant fields. The simulation was based on a given mechanical vibration with known parameters (frequency, amplitudes, and phases) and performed at different spatial positions and for different wave‐phase offsets. The phase accrual due to $B_{c}\left(x,y,z,t\right)$ and motion encoding gradients was obtained via numerical integration. Both the Hadamard motion encoding matrix H and the proposed encoding matrix M were implemented in the simulation. In both cases, decoding was performed and followed by a temporal Fourier transform to compare the retrieved mechanical vibration amplitudes and phases in the simulated volume at different spatial positions. The parameters of the numerical model were as follows: wave displacement amplitudes $\left[A_{x},A_{y},A_{z}\right]=\left[200\;\mathrm{\mu m},200\;\mathrm{\mu m},200\;\mathrm{\mu m}\right],$ wave displacement phases $\left[\theta_{x},\theta_{y},\theta_{z}\right]=\left[0\;\mathrm{rad},1.047\;\mathrm{rad},1.570\;\mathrm{rad}\right]$, motion encoding gradient amplitude =20mT/m, 9261 spatial positions uniformly sampled in a cube centered at isocenter with edge length $400\;\mathrm{mm},B_{0}=0.55\;T,$ motion encoding duration =6.7ms (we neglected gradient ramp up and ramp down times), mechanical frequency =60Hz, and four wave‐phase offsets. The numerical simulation was performed in MATLAB R2023b (The Math Works, 2023).

### 2.3 Phantom experiments

The proposed encoding matrix M is incorporated into a previously published 3D MRE sequence^20^ extending the sequence from four to six measurements. Please note, that the motion encoding gradients are applied without any overlap with the imaging gradients of the sequence.

**FIGURE 1:**
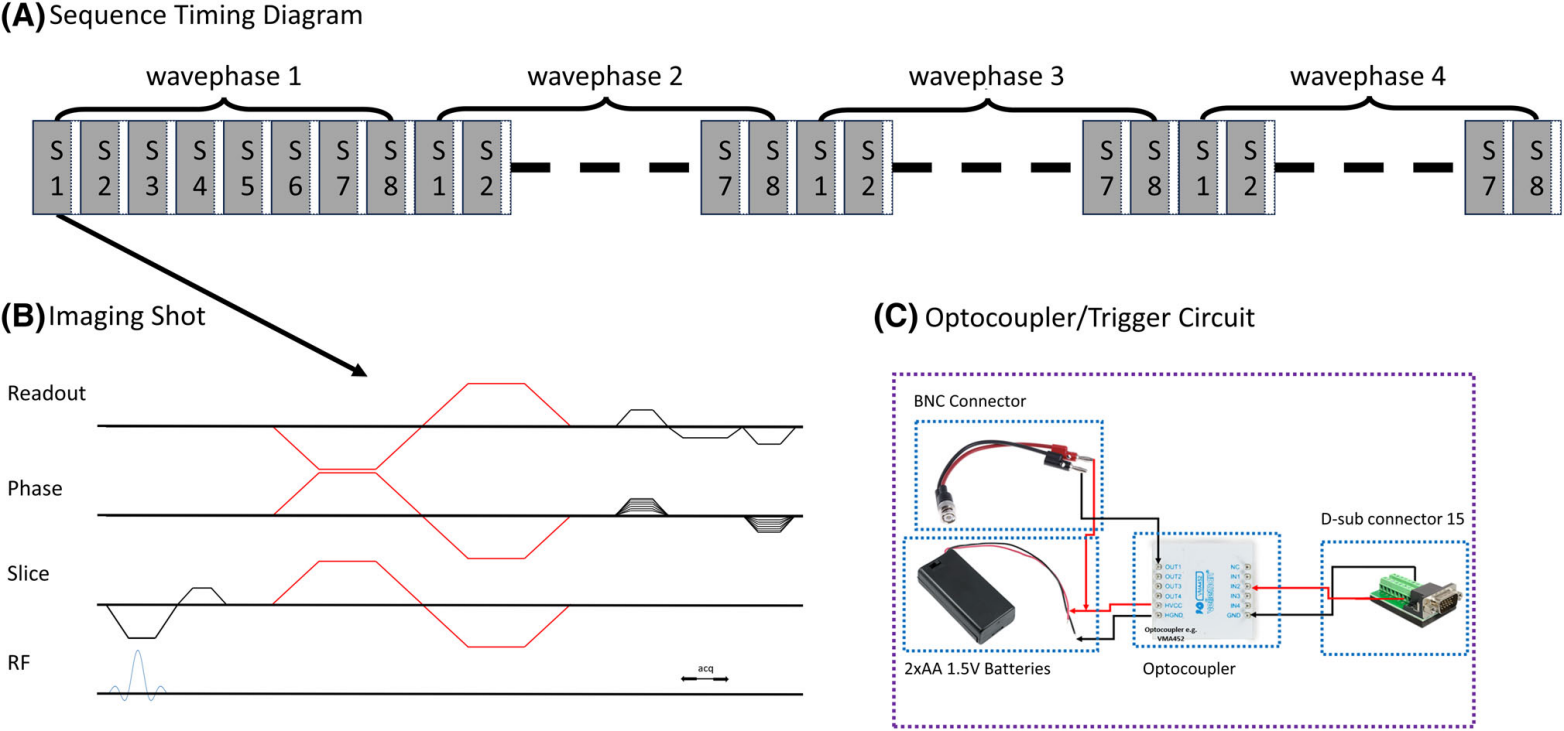
(A) The sequence timing diagram following a Ristretto MR elastography (MRE) acquisition scheme (Guenthner et al.^20^), shown for 8 slices (S1…8) and 4 wave offsets (wp1…4). The white spaces between the slice blocks indicate time delays to shift from one wave phase offset to another. (B) The imaging shot consists of a spoiled GRE sequence with the motion encoding gradient following the proposed extended encoding matrix. (C) The trigger circuit where an optocoupler was used to maintain electrical isolation between the MR system and the MRE device.

The extended 3D MRE sequence (Figure 1A,B) is implemented on a 0.55 T system (MAGNETOM Free.Max, Siemens Healthineers AG) with a 12‐channel head coil. Two phantom experiments are conducted: (1) an experiment without mechanical excitation to verify that M is solving for the cross terms. Please note, that the first cross term $\left(G_{x}G_{z}\mathrm{xz}\right)$ varies with *x* and z, and the second cross term $\left(G_{y}G_{z}\mathrm{yz}\right)$ varies with y and z, both independent of the mechanical excitation. (2) An experiment with 60 Hz mechanical excitation^21^ to verify that the phase accrual because of $B_{c}$ is constant with respect to the acquired mechanical wave phase offsets and can be filtered out using temporal Fourier transform. The phantom used is a candle wax phantom shifted away from the isocenter of the bore in the z direction (100 mm) to increase the apparent effect of the concomitant fields.

The imaging parameters of the extended 3D MRE sequence were as follows: 8 slices, 4 mm isotropic resolution, a 96 × 64 acquisition matrix, flip angle = 25°, in‐plane GRAPPA acceleration factor of 2 resulting in a FOV of 386 × 256 × 32 mm^3^, TR = 18.24 ms, TE = 12.90 ms (in‐phase condition at 0.55 T), receiver bandwidth = 180 Hz/px. The motion encoding gradients of the extended 3D MRE sequence followed the proposed 6 × 6 encoding matrix M and had a duration of 6.7 ms, an amplitude of 20 mT/m, and an encoding efficiency of 17.2 rad/mm.

The 3D MRE sequence was also implemented on a 3 T system (Biograph mMR, Siemens Healthineers AG) to compare concomitant field effects across field strengths. The imaging parameters at 3 T were similar to those used at 0.55 T, with the exception of TE (7.38 ms), TR (9.38 ms), and receiver bandwidth (700 Hz/px).

### 2.4 Healthy volunteer experiments—The feasibility of hepatic 3D MRE at 0.55 T

This proof‐of‐concept study was conducted in March 2023 and was approved by the local institutional review board. Five healthy volunteers were enrolled after signing informed consent and were scanned at 60 Hz mechanical excitation using the gravitational transducer^21^ with a previously published multiple breath‐hold hepatic 3D MRE sequence^20^ implemented on a 0.55 T system (MAGNETOM Free.Max, Siemens Healthineers AG) with a 6‐channel body coil and a 9‐channel spine coil in the supine position. The synchronization of the gravitational transducer with the 3D MRE sequence was achieved using transistor‐transistor logic signals sent by the 0.55 T system where an optocoupler was used to maintain electrical isolation as shown in Figure 1C. The total acquisition time at 0.55 T was 84 s preformed in four breath‐holds of 21 s each.

In addition, the five healthy volunteers underwent a hepatic 3D MRE scan on a 3 T system (Biograph mMR, Siemens Healthineers AG) using a previously published multiple breath‐hold hepatic 3D MRE sequence^20^ to compare the retrieved viscoelastic parameters on 0.55 T with those retrieved on 3 T. The total acquisition time at 3 T was 48 s preformed in four breath‐holds of 12 s each.

The imaging parameters for both 0.55 T and 3 T were similar to those used in the phantom experiments and a four‐measurement Hadamard encoding scheme was used in all healthy volunteer experiments.

### 2.5 Data processing and analysis

All image processing was performed by an operator with 6 years of experience in MRE. Decoding was applied to the acquired MRE phase images via the inverse of M before coil combination. The obtained phase images were subsequently unwrapped using a minimum cost flow technique.^22^ Afterward, pixelwise temporal Fourier transform was applied to extract the 3D displacement field. Finally, the viscoelastic parameters were retrieved using the curl‐operator method^23^ and solving for the complex *k* vector of the wave propagation.^3^

The phantom experiments were assessed by visual inspection and comparison of the unwrapped phase images of $\varphi_{U_{x}}$, $\varphi_{\mathrm{xz}}$, and $\varphi_{\mathrm{yz}}$ for a single slice and one wave phase offset. In addition, the amplitudes $|U_{\mathrm{xz}}|$ and $|U_{\mathrm{yz}}|$ of the apparent displacement fields encoded in $\varphi_{\mathrm{xz}}$ and $\varphi_{\mathrm{yz}}$, respectively, were calculated in a manually drawn region of interest (ROI) and the mean values are compared with the mean value of the total amplitude ($A_{\mathrm{tot}})$ of the mechanical vibration at 60 Hz in the phantom in the same ROI. Furthermore, the mean value of |G*| [kPa], Cs [m/s], and *G*″ [kPa] in the phantom was calculated at 0.55 T and 3 T for the proposed 6 × 6 proposed encoding and for the 4 × 4 Hadamard encoding that was obtained by selecting the first four steps of the 6 × 6 encoding scheme.

The viscoelastic maps of the healthy volunteers were calculated by averaging the central four slices, and the mean values of |G*| [kPa], Cs [m/s], and *G*″ [kPa] were reported from ROIs in the liver defined away from large vessels and organ edges. The comparison between the viscoelastic parameters retrieved on 0.55 T and 3 T was assessed using the Bland–Altman method by studying the mean differences and the 95% limits of agreement, as well as a paired sample *t* test where *p* < 0.05 was considered to indicate a statistically significant result. All data processing was preformed using KIR software (v2021, King's College London). Lastly, noise in parallel imaging is spatially variable,^24^ therefore, instead of estimating SNR, an apparent SNR comparison^2^ was performed between 0.55 T and 3 T in the healthy volunteers to evaluate the relative SNR decrease at 0.55 T. Apparent SNR was estimated at 0.55 T and 3 T in the magnitude images for the first motion encoding step and first wave phase offset as the ratio of the mean value of the pixels in a ROI in the liver and the SD of the pixels in a region of interest in the background.

## 3 RESULTS

### 3.1 Numerical simulations

For the simulated encoding matrix *M*, the amplitudes $|U_{\mathrm{xz}}|$ and $|U_{\mathrm{yz}}$| of the apparent displacement fields encoded in $\varphi_{\mathrm{xz}}$ and $\varphi_{\mathrm{yz}}$ relative to $A_{\mathrm{tot}}$ are less than 1% (Table 1). In addition, there is no significant difference (*p* < 0.005) between the amplitudes $A_{x}$, $A_{y}$, and $A_{z}$ and the phases $\theta_{x}$, $\theta_{y}$, and $\theta_{z}$ retrieved with Hadamard encoding matrix H and the amplitudes and phases retrieved with the proposed encoding matrix M (Table 2). All the retrieved mechanical vibration parameters were very close to the simulated ground truth values (Table 2).

**TABLE 1:** Mean values of $A_{\mathrm{tot}}$ the total amplitude of the 3D mechanical vibration in μm, and $\left|U_{\mathrm{xz}}\right|,\left|U_{\mathrm{yz}}\right|$ the amplitudes in μm of the apparent displacements encoded in $\varphi_{\mathrm{xz}}$ and $\varphi_{\mathrm{yz}}$, respectively, averaged over the entire simulated volume

Simulation	$A_{\mathrm{tot}}$ [μm]	$\left|U_{\mathrm{xz}}\right|$ [μm]	$\left|U_{\mathrm{yz}}\right|\;\left[\mathrm{\mu m}\right]$	$\frac{\left|U_{\mathrm{xz}}\right|}{A_{\mathrm{tot}}}$	$\frac{\left|U_{\mathrm{yz}}\right|}{A_{\mathrm{tot}}}$
6 × 6 encoding	349.01 ± 1e−11	0.80 ± 0.30	0.74 ± 0.43	0.002 ± 0.001	0.002 ± 0.001
				Max = 0.0041	Max = 0.0055

*Notes*: The ratios of $\left|U_{\mathrm{xz}}\right|,\left|U_{\mathrm{yz}}\right|$ with respect to $A_{\mathrm{tot}}$ are also provided as mean ± SD along with the maximum values of these ratios over the entire simulated volume.

Abbreviation: Max, maximum.

**TABLE 2:** Mean values of the amplitude $A_{x}$, $A_{y}$, $A_{z}$, and the phases $\theta_{x}$, $\theta_{y}$, and $\theta_{z}$ retrieved from the numerical simulation for the 4 × 4 Hadamard encoding and 6 × 6 proposed encoding

Simulation	$A_{x}$ [μm]	$A_{y}$ [μm]	$A_{z}$ [μm]	$\theta_{x}$ [rad]	$\theta_{y}$ [rad]	$\theta_{z}$ [rad]
4 × 4 encoding	201.51 ± 0.80	201.51 ± 0.61	201.51 ± 9e−12	4e−6 ± 0.0015	1.047 ± 0.003	1.571 ± 6e−14
6 × 6 encoding	201.51 ± 9e−12	201.51 ± 9e‐12	201.51 ± 9e−12	−2e−16 ± 9e−15	1.047 ± 1e−14	1.571 ± 6e−14

*Notes*: All mean values are calculated over the entire simulated volume. It is noted that the retrieved $A_{z}$ and $\theta_{z}$ are mathematically identical between the two encoding schemes (the decoding for these parameters is solely relying on the first four measurements, which are identical for both encoding schemes).

### 3.2 Phantom experiments

**FIGURE 2:**
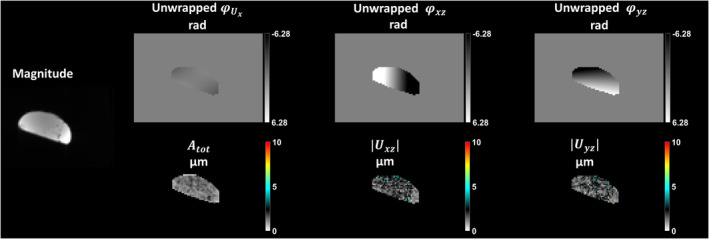
Phantom experiment without mechanical excitation at 0.55 T. The magnitude image was calculated by averaging the central four slices for all motion encoding steps and wave phase offsets. The unwrapped phases ($\varphi_{U_{x}}$, $\varphi_{\mathrm{xz}}$, and $\varphi_{\mathrm{yz}}$) in radians are shown for a single slice and one wave phase offset after decoding. $A_{\mathrm{tot}}$ is the total amplitude of the 3D mechanical vibration in μm, and $\left|U_{\mathrm{xz}}\right|,\left|U_{\mathrm{yz}}\right|$ are the amplitudes in μm of the apparent displacements encoded in $\varphi_{\mathrm{xz}}$ and $\varphi_{\mathrm{yz}}$, respectively, averaged over the central four slices.

The results of the phantom experiments without mechanical excitation at 0.55 T are shown in Figure 2. There is no spatial variation in $\varphi_{U_{x}}$. However, we can observe a spatial variation in x (left–right direction) in $\varphi_{\mathrm{xz}}$ and a spatial variation in y (posterior–anterior direction) in $\varphi_{\mathrm{yz}}$. The spatial variation observed suggests that the proposed encoding matrix M is solving for the “cross” terms. The amplitudes $|U_{\mathrm{xz}}|$ and $|U_{\mathrm{yz}}|$ of the apparent displacement fields encoded in $\varphi_{\mathrm{xz}}$ and $\varphi_{\mathrm{yz}}$ are very low (1.73 ± 0.60 μm and 1.65 ± 0.59 μm, respectively), and $A_{\mathrm{tot}}$ is 1.25 ± 0.32 μm.

**FIGURE 3:**
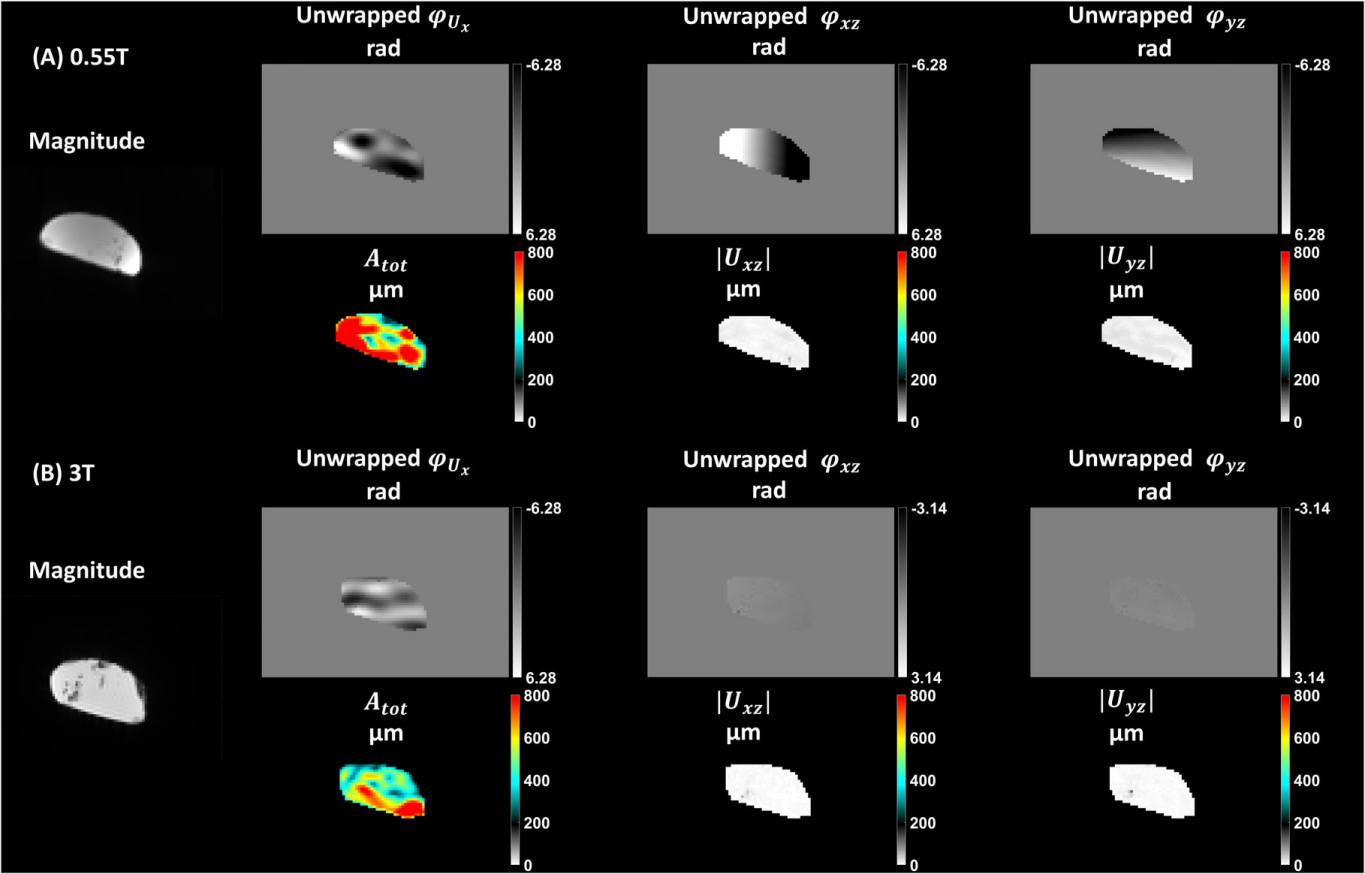
Phantom experiment with 60 Hz mechanical excitation at 0.55 T (A) and 3 T (B). The magnitude image is calculated by averaging the central four slices for all motion encoding steps and wave phase offsets. The unwrapped phases ($\varphi_{U_{x}}$, $\varphi_{\mathrm{xz}}$, and $\varphi_{\mathrm{yz}}$) in radians are shown for a single slice and one wave phase offset after decoding. $A_{\mathrm{tot}}$ is the total amplitude of the 3D 8mechanical vibration in μm, and $\left|U_{\mathrm{xz}}\right|,\left|U_{\mathrm{yz}}\right|$ are the amplitudes in μm of the apparent displacements encoded in $\varphi_{\mathrm{xz}}$ and $\varphi_{\mathrm{yz}}$, respectively, averaged over the central four slices. A different scaling is used for the “cross” term phase images of the phantom experiment at 3 T for improved visualization.

The results of the phantom experiment with mechanical excitation at 0.55 T and 3 T are shown in Figure 3. $\varphi_{U_{x}}$ varies with the mechanical excitation at 0.55 T and 3 T, however, $\varphi_{\mathrm{xz}}$ and $\varphi_{\mathrm{yz}}$ only vary spatially in x (left–right direction) and in y (posterior–anterior direction), respectively. The spatial variation of $\varphi_{\mathrm{xz}}$ and $\varphi_{\mathrm{yz}}$ is more apparent at 0.55 T than at 3 T that can be attributed to the inverse dependency of the concomitant fields on $B_{0}$.

The amplitudes $|U_{\mathrm{xz}}|$ and $|U_{\mathrm{yz}}$ |of the apparent displacement fields encoded in $\varphi_{\mathrm{xz}}$ and $\varphi_{\mathrm{yz}}$ at 0.55 T and 3 T are summarized in Table 3. The amplitudes of the apparent displacement fields relative to $A_{\mathrm{tot}}$ were similar across field strengths and of the order of 1% to 2%.

**TABLE 3:** Mean values of $A_{\mathrm{tot}}$ the total amplitude of the 3D mechanical vibration in μm, and $\left|U_{\mathrm{xz}}\right|,\left|U_{\mathrm{yz}}\right|$ the amplitudes in μm of the apparent displacements encoded in $\varphi_{\mathrm{xz}}$ and $\varphi_{\mathrm{yz}}$, respectively, averaged over the central four slices in phantom experiments at 0.55 T and 3 T.

Experiment	$A_{\mathrm{tot}}$ [μm]	$\left|U_{\mathrm{xz}}\right|$ [μm]	$\left|U_{\mathrm{yz}}\right|$ [μm]	$\frac{\left|U_{\mathrm{xz}}\right|}{A_{\mathrm{tot}}}$	$\frac{\left|U_{\mathrm{yz}}\right|}{A_{\mathrm{tot}}}$
0.55 T with vibration	690.67 ± 244.85	8.31 ± 4.57	11.45 ± 4.59	0.012 ± 0.008	0.017 ± 0.009
3 T with vibrations	516.25 ± 178.60	4.33 ± 3.73	4.84 ± 5.82	0.008 ± 0.008	0.009 ± 0.011

*Notes*: The ratios of $\left|U_{\mathrm{xz}}\right|,\left|U_{\mathrm{yz}}\right|$ with respect to $A_{\mathrm{tot}}$ are also provided.

The mean values of the retrieved |G*|, Cs, *G*″ in the phantom are shown in Table 4. The mean values were similar across field strengths and encoding schemes.

**TABLE 4:** Mean values of the magnitude of the complex shear modulus |G*| [kPa], the shear wave speed Cs [m/s], and the loss modulus *G*″ [kPa] obtained in the phantom experiments.

Experiment	$\left|G^{*}\right|$ [kPa]	|Cs| [m/s]	$\left|G^{\prime \prime }\right|$ [kPa]
0.55 T 6 × 6 encoding	4.42 ± 0.23	2.14 ± 0.1	1.32 ± 0.21
3 T 6 × 6 encoding	4.38 ± 0.23	2.13 ± 0.1	1.36 ± 0.22
0.55 T 4 × 4 Hadamard	4.40 ± 0.23	2.14 ± 0.1	1.48 ± 0.22
3 T 4 × 4 Hadamard	4.28 ± 0.23	2.13 ± 0.1	1.48 ± 0.24

*Notes*: All mean values are calculated over the central four slices. 6 × 6 encoding is the proposed encoding method and 4 × 4 Hadamard is the original 4 × 4 Hadamard encoding matrix.

### 3.3 Healthy volunteer experiments—The feasibility of hepatic 3D MRE at 0.55 T

All five healthy volunteers underwent successful MR imaging. Three of the five healthy volunteers were female, and the mean age and BMI were 30.0 ± 5.3 years, and 22.1 ± 1.8 kg/m^2^, respectively.

Mean values of the liver in the five healthy volunteers were 2.1 ± 0.3 kPa for |G*|, 1.5 ± 0.1 m/s for Cs, and 0.8 ± 0.1 kPa for *G*″ at 0.55 T, and 2.0 ± 0.2 kPa for |G*|, 1.5 ± 0.1 m/s for Cs, and 0.9 ± 0.1 kPa for *G*″ at 3 T reflecting good agreement between 3D MRE across the two field strengths.

**FIGURE 4:**
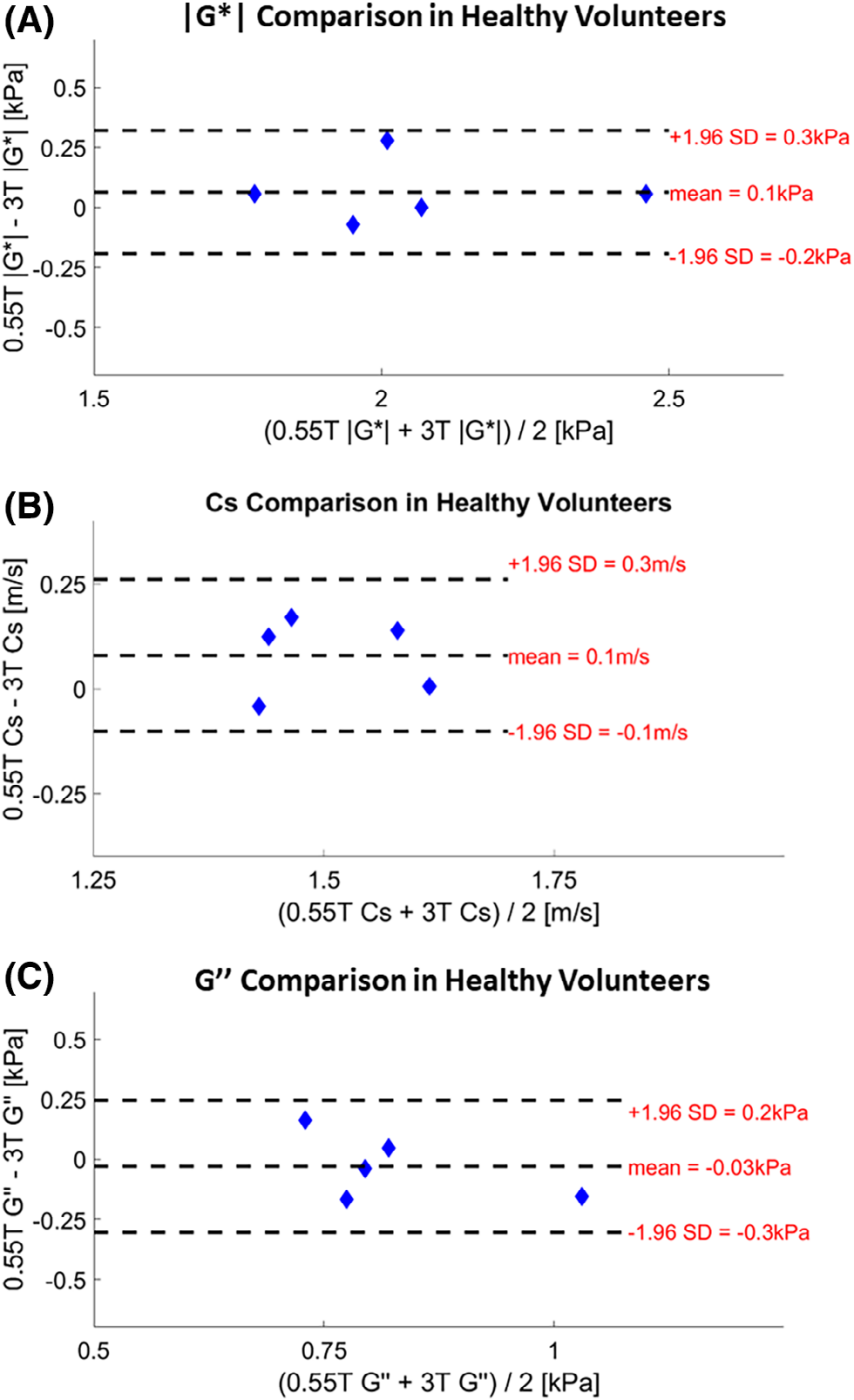
Bland–Altman plots for the comparison of the retrieved biomechanical parameters (A) |G*|, (B) Cs, and (C) G″) at 0.55 T and 3 T in healthy volunteers. The mean differences and the 95% limits of agreement are reported.

Bland–Altman plots of the agreement between 3D MRE at 0.55 T and 3D MRE at 3 T for the measurement of |G*|, Cs, and *G*″ are shown in Figure 4 (A‐C). Bland–Altman analysis shows mean differences of 0.1 kPa for |G*| (95% limits of agreement: −0.2,0.3), 0.1 m/s for Cs (95% limits of agreement: −0.1,0.3) and − 0.03 kPa for *G*″ (95% limits of agreement: −0.3,0.2), suggesting good agreement between 3D MRE viscoelastic parameters at 0.55 T and 3 T. Paired sample *t* tests indicated that there is no significant difference in |G*| (*t*‐value = 1.12, *p* = 0.33), Cs (*t*‐value = 1.96, *p* = 0.12), and *G*″ (*t*‐value = −0.6, *p* = 0.57) between 0.55 T and 3 T.

**FIGURE 5:**
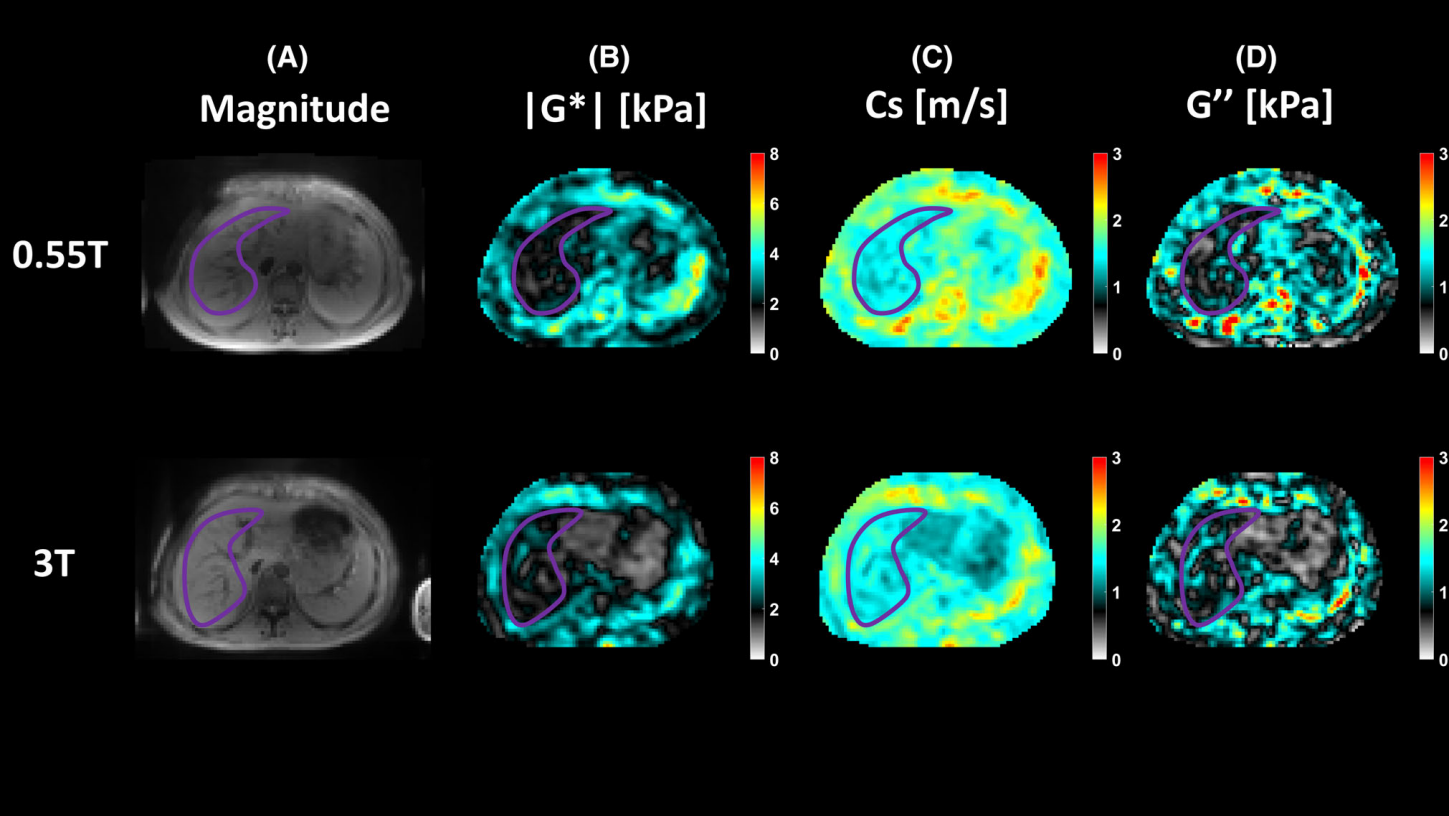
MR elastography results for a representative healthy volunteer at 0.55 T (top row) and 3 T (bottom room). The outlines of the liver are highlighted in purple. (A) Magnitude images, (B) the magnitude of the complex shear modulus |G*| [kPa], (C) the shear wave speed Cs [m/s], and (D) the loss modulus *G*″ [kPa]. All maps show average value over the central four slices.

The MRE images obtained from a representative healthy volunteer at 0.55 T and 3 T are shown in Figure 5. Visual inspection of the magnitude images shows very similar anatomical features at both field strengths and the calculated viscoelastic maps at both field strengths reflect good agreement.

The SNR analysis showed a 2.1‐fold decrease in the SNR of 3D MRE at 0.55 T in comparison with the 3D MRE at 3 T (mean relative SNR, 0.47 ± 0.11).

## 4 DISCUSSION

We characterized the effects of concomitant fields in MRE and confirmed that the effects are negligible using numerical simulations and phantom experiments at 0.55 T and 3 T, and this part of the work did not rely on organ‐specific assumptions. Additionally, we demonstrated the feasibility of hepatic 3D MRE at 0.55 T in healthy volunteers, showing good agreement between the retrieved biomechanical parameters at 0.55 T and 3 T.

Currently, the clinical standard for hepatic MRE is carried out on 1.5 and 3 T.^25^, ^26^, ^27^, ^28^ Our aim was to expand MRE to wide‐bore 0.55 T systems to accommodate liver patients with high BMI. High BMI is often associated with NAFLD, which has become an important public health crisis because of its potential progression into severe chronic liver disease.^29^ Using 3D MRE allows for the early detection of liver inflammation in NAFLD patients,^2^, ^3^ a crucial aspect for patient management as the inflammatory process is reversible.^30^, ^31^ MRE at low field strength has been previously introduced.^9^, ^11^, ^32^ However, these studies focused on MRE in phantoms and tissue samples^9^, ^32^ and human extremity muscles^11^ and did not consider the effects of concomitant fields nor the application of 3D MRE in the liver.

In our study, we characterized the effects of concomitant fields in 3D MRE using a Taylor series expansion and verified that the temporal Fourier transform used in MRE reconstruction removes the phase errors because of the concomitant fields. The derivation is closely related to the work about concomitant field effects in phase contrast MRI^18^ where $\varphi_{c,\text{static}}$ has been studied and confirms that concomitant fields have significantly lower motion encoding effects compared to typically used motion encoding gradient strengths. Our analysis was based on a temporal Fourier transform applied on the measured encoded signals, while our reconstruction is applying the temporal Fourier transform on the decoded signals. However, the analysis still holds because the Fourier transform and the decoding are linear operators. We also proposed an encoding scheme to assess the apparent displacements encoded because of concomitant field terms in numerical simulations and in phantom experiments. Confirming the results of our numerical simulations, the amplitudes of these apparent displacements relative to total wave amplitude in the phantom experiments were close to the noise level and similar between 0.55 T and 3 T, and the viscoelastic parameters obtained using 6 × 6 and 4 × 4 decoding schemes were similar, suggesting that the measured apparent displacements are dominated by vibration and image noise.^33^ The encoding‐decoding scheme supposes a perfect stability for exact cancellation of effects unrelated to concomitant fields, but this is difficult to realize in practice, therefore, we put on a lot of emphasis on ensuring a high stability of our phantom experimental setup to avoid phantom bulk motion.

Despite the observed decrease in SNR at 0.55 T in comparison with 3D MRE at 3 T, and therefore, its impact on PNR,^8^ we successfully demonstrated the feasibility of 3D hepatic MRE at 0.55 T. We used the inherent averaging of the Hadamard motion encoding scheme^12^ to compensate the decrease in PNR at 0.55 T, which did not compromise the quality of the retrieved biomechanical parameters. In addition, T1 relaxation times in the liver are shorter at 0.55 T than at 3 T,^34^ which, combined with a longer TR and lower receiver bandwidth at 0.55 T, may have mitigated the lower SNR and PNR associated with the lower static magnetic field. The averaging effect of the four‐step Hadamard encoding scheme results in a theoretical twofold PNR increase. Combined with the fourfold decrease in the used receiver bandwidth at 0.55 T, this would result in a total fourfold increase in PNR. Assuming that PNR is linearly proportional to the static magnetic field, it is expected that the 0.55 T results would be equivalent to 1.5 T, although a full comparison between 0.55 T and 1.5 T is beyond the scope of this study.

Our results show that the viscoelastic parameters measured at 0.55 T are similar to those measured at 3 T despite the observed bias: 0.1 kPa for |G*| (95% limits of agreement: −0.2,0.3), 0.1 m/s for Cs (95% limits of agreement: −0.1,0.3), and −0.03 kPa for *G*″ (95% limits of agreement: −0.3,0.2). Previous work has shown mean differences of up to 0.5 kPa in the magnitude of the complex‐valued shear modulus (|G*| [kPa]) across field strengths (1.5 T and 3 T), and MR vendors.^35^, ^36^, ^37^, ^38^ However, discrepancies of this magnitude are unlikely to affect the primary clinical utility of MRE in differentiating between high‐grade and low‐grade fibrosis or no fibrosis.^36^

This study has several limitations. We only considered the case where Hadamard motion encoding gradients are not overlapping with any other imaging gradients, which restricts the total duration available for motion encoding gradients. However, the first in‐phase echo at 0.55 T is achieved at a relatively long TE (12.9 ms), which provides sufficient time for motion encoding. It is also worth noting that Hadamard motion encoding with overlapping gradients might lead to restriction of the gradient amplitude used for motion encoding in order not to exceed the maximum gradient amplitude and slew rate specified by the system. A further limitation of this study is that we studied the case of bipolar motion‐encoding gradients only, and we did not consider flow‐compensated gradients. The motion encoding gradients along the different axes were considered to be applied at the same time with respect to the wave phase‐offset, therefore, the analysis would not apply to MRE methods such as SLIM‐MRE^39^ where these motion‐encoding gradients are applied along all physical axes simultaneously, but shifted with respect to each other.

Furthermore, in the phantom experiments, the 6 × 6 encoding scheme is able to resolve only the cross‐terms of the concomitant fields, whereas the self‐squared terms cannot be separated from other sources of constant phase errors such as motion encoding because of imaging gradients. In addition, the phantom experiments were only conducted with a shift in the z direction rather than a shift in x, y, and z.

A further limitation of this study is that the breath‐hold duration is too long to be tolerated by all patients in the clinic. Techniques such as simultaneous multi‐slice excitation^2^, ^40^ and compressed sensing^41^ may be beneficial to reduce the breath‐hold duration. Furthermore, this study examined the effects of the concomitant fields only in phantom experiments. Lastly, this is only a first attempt at comparing viscoelastic parameters between 0.55 T and 3 T in a small healthy volunteer group that had a BMI that falls within the healthy range (22.4 ± 2.1 kg/m^2^) and a clinical study in a large NAFLD patient cohort is warranted to validate this technique at 0.55 T.

In conclusion, we found that the effects of concomitant fields on 3D Hadamard‐encoded MRE at 0.55 T are negligible. Moreover, to our knowledge, this work is the first attempt to address hepatic 3D MRE at 0.55 T, which may pave the pathway for broader adoption of 3D MRE in the clinic.

## CONFLICT OF INTEREST STATEMENT

The first author is a PhD candidate, and their university tuitions fees are covered by Siemens Healthineers. In addition, the first author is a Siemens Healthineers employee.

## Supporting information


**Material S1.** Analytical solution of the phase accrual because of $B_{c}$ of a spin isochromat undergoing a periodic sinusoidal motion.

**Material S2.** $\left(\mu_{\mathrm{x,wp}2}-\mu_{\mathrm{x,wp}1}\right)$ relative to $\left(x_{\mathrm{wp}2}-x_{\mathrm{wp}1}\right)$.
