# Supplementary material for: 3D MR elastography at 0.55 T: Concomitant field effects and feasibility
Source: Magn Reson Med. 2024 Nov 25;93(4):1602–14. doi: 10.1002/mrm.30377 (PMC11782726; doi:10.1002/mrm.30377)
Supplement: Supplementary file 1 — Material S1. Analytical solution of the phase accrual because of Bc of a spin isochromat undergoing a periodic sinusoidal motion. [file MRM-93-1602-s002.docx]

**Supplementary Material 1:** Analytical solution of the phase accrual due to $B_{c}$ of a spin isochromat undergoing a periodic sinusoidal motion.

Let us assume that a spin isochromat is subject to a mono-frequency mechanical excitation in steady-state. Thus, the spin isochromat experiences a periodic sinusoidal motion $r_{x}, r_{y}, r_{z}$ in $x_{,}y,$and $z$respectively as follows:

$$r_{x}=A_{x}sin\left( \omega t+\theta_{x}+{wp}_{k} \right)+x_{0}$$

$$r_{y}=A_{y}sin\left( \omega t+\theta_{y}+{wp}_{k} \right)+y_{0}$$

$$r_{z}=A_{z}sin\left( \omega t+\theta_{z}+{wp}_{k} \right)+z_{0}$$

( A 1 )

Where $A_{x}$, $A_{y}$, and $A_{z}$ are the local displacement amplitudes, $\omega$ is the frequency of the motion, $\theta_{x}$, $\theta_{y}$*,* and $\theta_{z}$ are the local wave phases, ${wp}_{k}$ is the k-th wave phase offset (k=1,2,3,…), and $x_{0}$, $y_{0},$ and $z_{0}$ are the initial position of the spin.

The phase accrual $\varphi_{enc}$ due to motion encoding gradients $G_{x}, G_{y},$ and $G_{z}$over a time period T of a spin undergoing a periodic sinusoidal motion is then given by the following integral:

$$\varphi_{enc}= \gamma\int_{0}^{T} {(r}_{x}G_{x}+ r_{y}G_{y}+r_{z}G_{z})dt$$

$$\varphi_{enc}= \frac{4\gamma\sin\left( \frac{T\omega}{4} \right)^{2}}{\omega}\left\{ A_{x}G_{x}\cos\left( \theta_{x}+{wp}_{k}+\frac{T\omega}{2} \right)+A_{y}G_{y}\cos\left( \theta_{y}+{wp}_{k}+\frac{T\omega}{2} \right)+A_{z}G_{z}\cos\left( \theta_{z}+{wp}_{k}+\frac{T\omega}{2} \right) \right\}$$

( A 2 )

The phase accrual $\varphi_{c}$ due to $B_{c}$ of a spin isochromat undergoing a periodic sinusoidal motion over a time period T is given by the following integral:

$$\varphi_{c}= \gamma\int_{0}^{T} B_{c}(r_{x},r_{y},r_{z},t)dt= \gamma\int_{0}^{T} \frac{1}{2B_{0}}\left( G_{x}^{2}{r_{z}}^{2}+G_{y}^{2}{r_{z}}^{2}+ G_{z}^{2}\frac{{r_{x}}^{2}+{r_{y}}^{2}}{4}- G_{x} G_{z}r_{x}r_{z}- G_{y} G_{z}r_{y}r_{z} \right)dt$$

$$\begin{aligned} =\frac{A_{z}^{2}G_{x}^{2}T\gamma}{4B_{0}}+&\frac{A_{z}^{2}G_{y}^{2}T\gamma}{4B_{0}}+\frac{A_{x}^{2}G_{z}^{2}T\gamma}{16B_{0}}+\frac{A_{y}^{2}G_{z}^{2}T\gamma}{16B_{0}}+\frac{G_{z}^{2}Ty_{0}^{2}\gamma}{8B_{0}}+\frac{G_{z}^{2}Ty_{0}^{2}\gamma}{8B_{0}}- \\ &\frac{G_{x}G_{z}Tx_{0}z_{0}\gamma}{2B_{0}}+\frac{G_{y}G_{z}Ty_{0}z_{0}\gamma}{2B_{0}}+\frac{G_{x}^{2}Tz_{0}^{2}\gamma}{2B_{0}}+\frac{G_{y}^{2}Tz_{0}^{2}\gamma}{2B_{0}}-\frac{A_{x}A_{z}G_{x}G_{z}T\gamma\mathrm{Cos}[\theta_{x}-\theta_{z}]}{4B_{0}}- \\ &\frac{A_{y}A_{z}G_{y}G_{z}T\gamma\mathrm{Cos}[\theta_{y}-\theta_{z}]}{4B_{0}}+\frac{A_{x}G_{z}^{2}x_{0}\gamma\mathrm{Cos}[\theta_{x}+wp_{k}]}{4B_{0}\omega}-\frac{A_{x}G_{x}G_{z}z_{0}\gamma\mathrm{Cos}[\theta_{x}+wp_{k}]}{2B_{0}\omega}+ \\ &\frac{A_{y}G_{z}^{2}y_{0}\gamma\mathrm{Cos}[\theta_{y}+wp_{k}]}{4B_{0}\omega}-\frac{A_{y}G_{y}G_{z}z_{0}\gamma\mathrm{Cos}[\theta_{y}+wp_{k}]}{2B_{0}\omega}-\frac{A_{z}G_{x}G_{z}x_{0}\gamma\mathrm{Cos}[\theta_{z}+wp_{k}]}{2B_{0}\omega}- \\ &\frac{A_{z}G_{y}G_{z}y_{0}\gamma\mathrm{Cos}[\theta_{z}+wp_{k}]}{2B_{0}\omega}+\frac{A_{z}G_{x}^{2}z_{0}\gamma\mathrm{Cos}[\theta_{z}+wp_{k}]}{B_{0}\omega}+\frac{A_{z}G_{y}^{2}z_{0}\gamma\mathrm{Cos}[\theta_{z}+wp_{k}]}{B_{0}\omega}- \\ &\frac{A_{x}G_{z}^{2}x_{0}\gamma\mathrm{Cos}[\theta_{x}+T\omega+wp_{k}]}{4B_{0}\omega}+\frac{A_{x}G_{x}G_{z}z_{0}\gamma\mathrm{Cos}[\theta_{x}+T\omega+wp_{k}]}{2B_{0}\omega}- \\ &\frac{A_{y}G_{z}^{2}y_{0}\gamma\mathrm{Cos}[\theta_{y}+T\omega+wp_{k}]}{4B_{0}\omega}+\frac{A_{y}G_{y}G_{z}z_{0}\gamma\mathrm{Cos}[\theta_{y}+T\omega+wp_{k}]}{2B_{0}\omega}+ \\ &\frac{A_{z}G_{x}G_{z}x_{0}\gamma\mathrm{Cos}[\theta_{z}+T\omega+wp_{k}]}{2B_{0}\omega}+\frac{A_{z}G_{y}G_{z}y_{0}\gamma\mathrm{Cos}[\theta_{z}+T\omega+wp_{k}]}{2B_{0}\omega}- \\ &\frac{A_{z}G_{x}^{2}z_{0}\gamma\mathrm{Cos}[\theta_{z}+T\omega+wp_{k}]}{B_{0}\omega}-\frac{A_{z}G_{y}^{2}z_{0}\gamma\mathrm{Cos}[\theta_{z}+T\omega+wp_{k}]}{B_{0}\omega}- \\ &\frac{A_{x}^{2}G_{z}^{2}\gamma\mathrm{Cos}[2\theta_{x}+T\omega+2wp_{k}]\mathrm{Sin}[T\omega]}{16B_{0}w}-\frac{A_{y}^{2}G_{z}^{2}\gamma\mathrm{Cos}[2\theta_{y}+T\omega+2wp_{k}]\mathrm{Sin}[T\omega]}{16\mathrm{BO}w}- \\ &\frac{A_{z}^{2}G_{x}^{2}\gamma\mathrm{Cos}[2\theta_{z}+T\omega+2wp_{k}]\mathrm{Sin}[T\omega]}{4B_{0}\omega}-\frac{A_{z}^{2}G_{y}^{2}\gamma\mathrm{Cos}[2\theta_{z}+T\omega+2wp_{k}]\mathrm{Sin}[T\omega]}{4B_{0}\omega}- \\ &\frac{A_{x}A_{z}G_{x}G_{z}\gamma\mathrm{Sin}[\theta_{x}+\theta_{z}+2wp_{k}]}{8B0w}-\frac{A_{y}A_{z}G_{y}G_{z}\gamma\mathrm{Sin}[\theta_{y}+\theta_{z}+2wp_{k}]}{8BOw}+ \\ &\frac{A_{x}A_{z}G_{x}G_{z}\gamma\mathrm{Sin}[\theta_{x}+\theta_{z}+2T\omega+2wp_{k}]}{8B_{0}\omega}+\frac{A_{y}A_{z}G_{y}G_{z}\gamma\mathrm{Sin}[\theta_{y}+\theta_{z}+2T\omega+2wp_{k}]}{8B_{0}\omega} \\ & \end{aligned}$$

( A 3 )

When comparing the terms in of $\varphi_{c}$ with the terms of $\varphi_{enc}$ , most terms are negligible except:

$$\varphi_{c} \approx\frac{\gamma T}{2B_{0}}\left( G_{x}^{2}z_{0}^{2}+G_{y}^{2}z_{0}^{2}+ G_{z}^{2}\frac{x_{0}^{2}+y_{0}^{2}}{4}- G_{x}G_{z}x_{0}z_{0}- G_{y}G_{z}y_{0}z_{0} \right)$$

( A 4 )

We can note that Eq A 4 is equivalent to the equation of $\varphi_{c, static}$ shown previously in the manuscript (Eq 17). The terms of the integrals were evaluated using Mathematica (Wolfram Research, Inc.).
