# Supplementary material for: 3D MR elastography at 0.55 T: Concomitant field effects and feasibility
Source: Magn Reson Med. 2024 Nov 25;93(4):1602–14. doi: 10.1002/mrm.30377 (PMC11782726; doi:10.1002/mrm.30377)
Supplement: Supplementary file 2 — Material S2. μx,wp2−μx,wp1 relative to xwp2−xwp1. [file MRM-93-1602-s001.docx]

**Supplementary Material 2:** Analysis of order of magnitude of $\left( \mu_{x,wp2}-\mu_{x,wp1} \right)$ relative to $\left( x_{wp2}-x_{wp1} \right)$ .

Let us consider a sinusoidal vibration in $x$ denoted $A_{x}ⅇ^{i\left( \omega t+\theta_{x} \right)}$ in complex notation to facilitate the derivation. Then we can write:

$$\frac{\left( \mu_{x,wp2}-\mu_{x,wp1} \right)}{\left( x_{wp2}-x_{wp1} \right)}= \frac{\left( A_{x}ⅇ^{i\left( \frac{\omega T}{2}+\theta_{x}+\Phi\right)}-A_{x}ⅇ^{i\left( \theta_{x}+\Phi\right)} \right)- \left( A_{x}ⅇ^{i\left( \frac{\omega T}{2}+\theta_{x} \right)}-A_{x}ⅇ^{i\left( \theta_{x} \right)} \right)}{\left( A_{x}ⅇ^{i\left( \Phi+\theta_{x} \right)}- A_{x}ⅇ^{i\left( \theta_{x} \right)} \right)}$$

Where $\Phi$ is the phase introduced between wave phase offsets wp1 and wp2 and T is the total duration of the motion encoding gradient.

This can be simplified to:

$$\frac{\left( \mu_{x,wp2}-\mu_{x,wp1} \right)}{\left( x_{wp2}-x_{wp1} \right)}= ⅇ^{i\left( \frac{\omega T}{2} \right)}-1 =2 i ⅇ^{i\left( \frac{\omega T}{4} \right)} \sin\frac{\omega T}{4}$$

Taking the magnitude of the ratio we have:

$$\left| \frac{\left( \mu_{x,wp2}-\mu_{x,wp1} \right)}{\left( x_{wp2}-x_{wp1} \right)} \right|= 2 \left| \sin\frac{\omega T}{4} \right|=2 \left| \sin\frac{\pi f}{2f_{enc}} \right|$$

And taking the phase we obtain:

$$arg\left( \mu_{x,wp2}-\mu_{x,wp1} \right)=arg\left( x_{wp2}-x_{wp1} \right)+ \frac{\pi}{2} (1+ \frac{f}{f_{enc}})$$

Where $f$ and $f_{enc}$ are the linear vibration and motion encoding gradient frequencies, respectively $(f_{enc}=\frac{1}{T})$. We observe that for $f= f_{enc}$, the magnitude of the ratio is 2 and the phase shift is π.

We can therefore conclude that if $f_{enc}$ is reasonably close to $f$, which is the case for typical MRE experiments, then $\left( \mu_{x,wp2}-\mu_{x,wp1} \right)$ and $\left( x_{wp2}-x_{wp1} \right)$ have the same order of magnitude since both the ratio of the magnitudes vary smoothly with the ratio $\frac{f}{f_{enc}}$. For example, for$f_{enc}=4f$, the ratio of magnitudes is approximately equal to 0.8.
